# Thickness Dependent Nanostructural, Morphological, Optical and Impedometric Analyses of Zinc Oxide-Gold Hybrids: Nanoparticle to Thin Film

**DOI:** 10.1371/journal.pone.0144964

**Published:** 2015-12-22

**Authors:** Veeradasan Perumal, Uda Hashim, Subash C. B. Gopinath, R. Haarindraprasad, Wei-Wen Liu, P. Poopalan, S. R. Balakrishnan, V. Thivina, A. R. Ruslinda

**Affiliations:** 1 Biomedical Nano Diagnostics Research Group, Institute of Nano Electronic Engineering (INEE), Universiti Malaysia Perlis (UniMAP), Kangar, Perlis, Malaysia; 2 School of Microelectronic Engineering, University Malaysia Perlis (UniMAP), Kuala Perlis, Perlis, Malaysia; 3 School of Bioprocess Engineering, Universiti Malaysia Perlis (UniMAP), Arau, Perlis, Malaysia; Institute for Materials Science, GERMANY

## Abstract

The creation of an appropriate thin film is important for the development of novel sensing surfaces, which will ultimately enhance the properties and output of high-performance sensors. In this study, we have fabricated and characterized zinc oxide (ZnO) thin films on silicon substrates, which were hybridized with gold nanoparticles (AuNPs) to obtain ZnO-Au_x_ (x = 10, 20, 30, 40 and 50 nm) hybrid structures with different thicknesses. Nanoscale imaging by field emission scanning electron microscopy revealed increasing film uniformity and coverage with the Au deposition thickness. Transmission electron microscopy analysis indicated that the AuNPs exhibit an increasing average diameter (5–10 nm). The face center cubic Au were found to co-exist with wurtzite ZnO nanostructure. Atomic force microscopy observations revealed that as the Au content increased, the overall crystallite size increased, which was supported by X-ray diffraction measurements. The structural characterizations indicated that the Au on the ZnO crystal lattice exists without any impurities in a preferred orientation (002). When the ZnO thickness increased from 10 to 40 nm, transmittance and an optical bandgap value decreased. Interestingly, with 50 nm thickness, the band gap value was increased, which might be due to the Burstein-Moss effect. Photoluminescence studies revealed that the overall structural defect (green emission) improved significantly as the Au deposition increased. The impedance measurements shows a decreasing value of impedance arc with increasing Au thicknesses (0 to 40 nm). In contrast, the 50 nm AuNP impedance arc shows an increased value compared to lower sputtering thicknesses, which indicated the presence of larger sized AuNPs that form a continuous film, and its ohmic characteristics changed to rectifying characteristics. This improved hybrid thin film (ZnO/Au) is suitable for a wide range of sensing applications.

## Introduction

Recent advances in interdisciplinary research have provided insight into the detection of biomolecules and increased their potential applications as biomarkers [1,2]. Current states with the established facilities in the forefront of nanotechnology allow for the generation of multiple functionalized and miniature biosensors for continuous bioanalyte recognition [3–9]. A suitable substrate that supports the receptor in a biosensor determines the success and high performance of the biosensor. The substrate or thin film on the sensing surface is determined based on its suitability for the desired sensor. Different types of metal oxides have been proposed as substrates because they facilitate the redox reaction upon interaction with molecules, which is a strategy that is employed to improve the conductivity of a given sensor [10–15]. Several studies have demonstrated the suitability of zinc oxide (ZnO) as a substrate for biosensing applications [16–19]. Recently, Haarindraprasad et al. reported the advantages of ZnO as a good transducer for sensing applications [20].

ZnO is a well-known semiconductor material due to its large exciton-binding energy (60 meV). The interest in the use of ZnO is fueled by its use in electro-optical devices due to its direct, wide band gap. In addition, as a potential material for optoelectronic applications, ZnO has a fairly broad range of advantages due to its high electron mobility and suitability for simple fabrication of nanostructures, which potentially results in lower cost ZnO based devices. ZnO nanostructures, such as nanorods, nanowires and nanoflowers, may be suitable materials for integration with various sophisticated devices [21,22]. At extreme pHs, ZnO becomes a convenient material to interface with chemical and biological compounds and facilitate surface functionalization due to its biocompatibility and bio-affinity properties [19,20]. In addition to the previously mentioned properties of ZnO, additional factors make it preferable to other band-gap materials.

Novel ZnO nanostructures coupled with metal nanoparticles could improve the optical and electronic properties of the hybrid material. In addition, metal nanoparticles may improve the performance of hybrid nanostructures and enhance the band-edge emission at the expense of defect emission [23]. In this context, gold nanoparticles (AuNPs) appear to be the best counterpart for ZnO thin films because the AuNPs exhibit a higher electron affinity behavior and the highest Schottky barrier compared with that of other noble metals. In addition, Au complements ZnO in constructing nanocomposites because of its resistance to surface oxidation and because it is both catalytically active and optically sensitive [24–26]. Therefore, embedding AuNPs onto ZnO nanostructures enable a convenient, direct and selective binding process for biomolecules. Further, the presence of the defect emission from the ZnO nanostructure catalyzes the excitation activity of the surface plasmon of AuNPs where the defect emission and surface plasmon absorption are interconnected. The electron recombination in the valence band of the ZnO matrix is made possible due to the transfer of surface plasmon from the conduction band (CB) to the valence band (VB). Due to these appealing characteristics and their suitable optical properties, ZnO/Au has attracted much attention over the years [27,28]. In general, all of the reported Au doped ZnO thin film experiments employed different methods to deposit Au on the ZnO, which was fabricated by using various approaches. Films as thin as 5 to 900 nm and Au sizes varying from 2–10 nm have been reported. Furthermore, the photoluminescence spectra indicated that AuNP deposition can reduce the surface defects of a substrate [29–33].

This study provides novel insight into the ZnO/Au hybrid structure for the mass production for potential use as a biomaterial based on a complete and comprehensive characterization. This imposes a strict condition on the growth method as well as on the final hybrid material properties that is affordable to the general populace. Herein, we report a solution strategy for integrating AuNPs onto a ZnO thin film with varying thicknesses. Further, the structural, optical and electrical properties of the nanohybrid were investigated to determine the satisfactory Au sizes for hybridization on ZnO. In addition, we describe a simple spin coating technique for depositing ZnO with a focus on the thickness, grain size, defects and electron bonding state. A comprehensive characterization of the effects of the thickness and grain size on the optical and electrical behavior has been performed.

## Experiment Details

### Materials and Reagents

A ZnO seed solution sol-gel was prepared using zinc acetate dihydrate [Zn(CH_3_COO)_2_.2H_2_O] (98%; Sigma Aldrich) in ethanol (EtOH; 99.99%; J.T. Baker). Monoethanolamine (MEA; 99%; Merck) was used as a stabilizer. Hydrochloric acid (HCl; 37%; J.T. Baker), aqueous ammonia (NH_4_OH; 30%; J.T. Baker), hydrogen peroxide (H_2_O_2_; 30%; J.T. Baker) and deionized water were used to prepare the standard cleaning 1 (RCA1) and standard cleaning 2 (RCA2) solutions. A buffered oxide etchant (BOE; 6:1; J.T. Baker), negative photoresist (NR7-6000PY; Futurrex) and resist developer (RD6; Futurrex) were used for photolithography. The growth solution was prepared by mixing equal concentrations (25 mM) of zinc nitrate hexahydrate (99%; Sigma Aldrich) and hexamethylenetetramine (99%; Merck) in Deionized (DI) water.

### Fabrication of interdigitated electrodes (IDEs)

**Fig 1:**
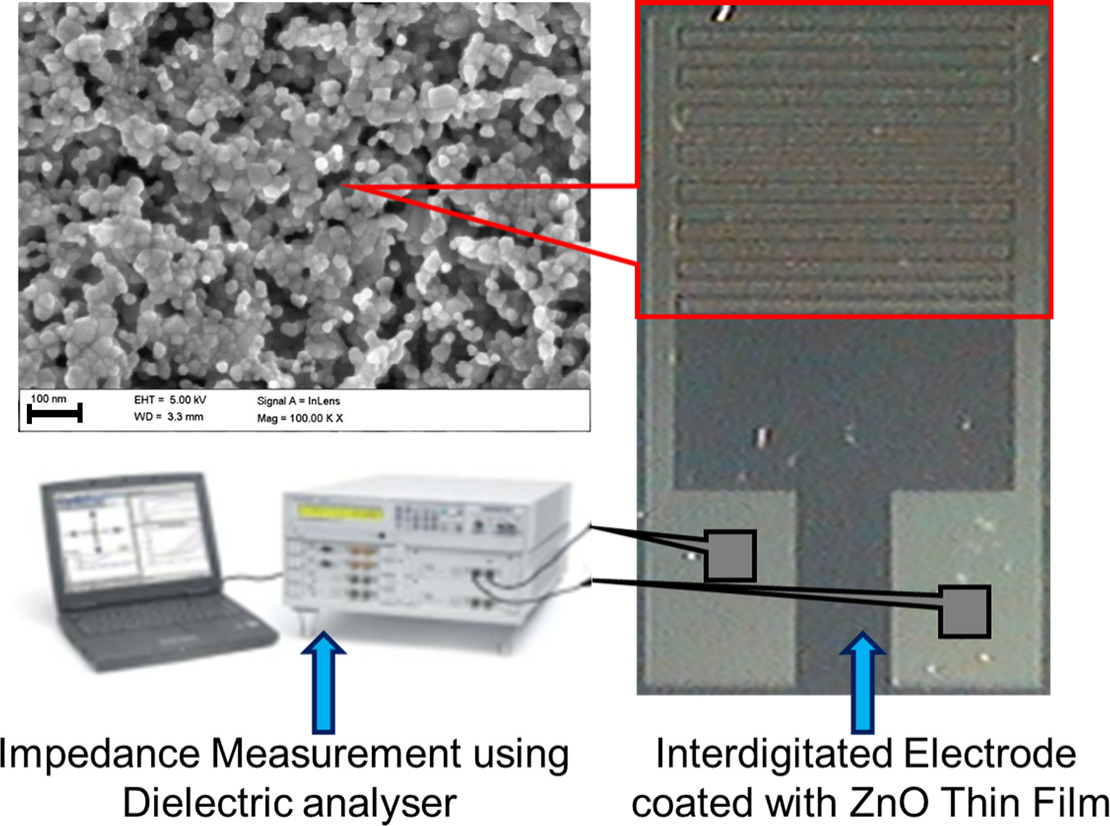
Au interdigitated electrode (IDE) for electrical measurement fabricated via simple and economical lithography route. Fabricated IDE array was coated with ZnO thin film, which was further sputtered with different thicknesses of Au.

A p-type silicon wafer was cleaned using RCA1, RCA2 and BOE to remove organic and inorganic contaminants as well as the native oxide layer on the wafer surface [34,35]. Next, the silicon wafer was rinsed and cleaned with deionized water. An approximately 200 nm thick SiO_2_ layer was produced on the clean wafer surface using a wet oxidation furnace. Using a conventional lithography process, an IDE device that was 7 *mm x* 5 *mm* in size patterned using negative resists (NR7-6000PY) on the SiO_2_/Si substrate. A thermal evaporator (Auto 306 thermal evaporator; Edwards High Vacuum International, Wilmington, MA, USA) was used to deposit a Titanium/Au (500/3000 Å) layer on the SiO_2_/Si substrate and patterned via a lift-off process [36]. Eventually, the negative photoresist sacrificial layer that formed was removed using acetone. In this study, IDE with 16 fingers was fabricated, where the width and length of each finger was 0.1 and 3.9 mm, respectively, and the spacing between the two adjacent fingers was 0.1 mm. The fabricated IDE are shown in Fig 1.

### Preparation of ZnO thin films (ZnO-TFs)

ZnO-TFs were prepared using a spin coating technique as follows: 8.78 g of Zn(CH_3_COO)_2_.2H_2_O were dissolved in 200 ml of ethanol (ZnO seed solution sol-gel). The concentration of ZnO was maintained at 0.2 M. Then, the mixed solution was vigorously stirred with a magnetic stirrer at 60°C for 30 min. The stabilizer (MEA) was added drop-wise to the ZnO solution with constant stirring for 2 h. Finally, the transparent and homogenous solution was stored for aging at room temperature. The aged ZnO sol-gel was deposited on the IDE device using a spin coating technique at a speed of 3000 rpm for 20 s. The deposition process of the seed layer was repeated 3 times to produce a thicker ZnO thin film. For each deposition process, the coated ZnO thin films were dried at 150°C for 20 min to remove the organic residuals that might exist on the ZnO thin films. Then, the coated ZnO thin films were annealed in a furnace under ambient air at 300°C for 2 h to yield highly crystallized ZnO.

### Preparation of Au sputtered ZnO nanocomposites

The ZnO thin film (ZnO-TF) was prepared using a spin coating technique and sputtering with Au to prepare the Au sputtered ZnO nanocomposites. To form the Au sputtered ZnO nanocomposites, 10, 20, 30, 40 and 50 nm Au wetting layers were physically deposited by a Sputter coater (EMS550X) with Au target and rotating stage. The detailed experimental conditions are as follows: electric current was maintained at 25 mA, for 2–8 min and the vacuum was maintained at 10^−2^ mbar. Under these conditions, we obtained Au sputtered ZnO thin films nanocomposites with various thicknesses.

### Microscopic nanoimaging

The morphology and structural properties of the Au sputtered ZnO nanocomposites samples were investigated using field emission scanning electron microscopy (FESEM; Carl Zeiss AG ULTRA55, Gemini). In addition, nanoscale imaging was employed using transmission electron microscopy (TEM) where small fraction/scraps of specimens were taken for this analysis. High-resolution transmission electron microscopy (HRTEM) images and selected area electron diffraction (SAED) patterns of the Au sputtered ZnO nanocomposites were recorded (PHILIPS, CM-200 TWIN) with an incident energy of 200 keV.

### Structural analysis

X-ray diffraction (XRD, Bruker D8, Bruker AXS, Inc., Madison, WI, USA) with Cu Kα radiation (λ = 1.54 Ǻ), Bragg-Brentano configuration with 2 decimal places angular accuracy goniometer was employed to study the crystallization and structural properties of the Au sputtered ZnO nanocomposites. The XRD pattern was recorded in the range of 30° to 60° and operating at a voltage of 40 kV and a current of 40 mA. The X-ray spectra peak analysis was performed using Diffraction plus 2003 version of the Eva 9.0 rev.0 software. The material composition, immobilization and hybridization were analyzed using X-ray photoelectron spectroscopy (XPS) (Omicron Dar400, Omicron, Germany). The chamber pressure was maintained at 2.4 e^−10^ Torr throughout the measurement. The obtained peaks were de-convoluted using the casaXPS software.

### Optical measurements

A UV-visible-near infrared spectrophotometer from PerkinElmer was used to study the optical properties of the Au sputtered ZnO thin film at room temperature. In addition, the optical and luminescence properties of the Au sputtered ZnO thin film were studied using photoluminescence (PL, Horiba Fluorolog-3, HORIBA Jobin Yvon Inc., USA). The PL spectra of the sample were recorded at different angles and positions to ensure that the result was not influenced by the heterogeneity of the sample.

### Impedance spectroscopy

The electrical measurements for the impedance spectroscopy analysis measurements were performed with a Novocontrol alpha high-frequency analyzer (Hundsangen, Germany). The impedance spectra of the real and imaginary parts of the impedance (i.e., Zs’ and Zs”, respectively) were obtained by sweeping the frequency from 1 to 100 MHz with an applied AC amplitude of 1 V RMS. All of the measurements were performed at room temperature. In addition, the electrical measurements of the current to voltage (I-V) were recorded using a Kiethley 6487 Picoammeter.

## Results and Discussion

Optimization of the ZnO thin film substrate is essential for tuning or altering the defect emission of tailored optoelectronic applications. The optimized ZnO, which has superior optical properties, can be employed for biomolecular recognition. Current results indicate that surface plasmon resonance (SPR) is a potential route for controlling the band emission of ZnO nanostructures. In addition, a ZnO host matrix with incorporated Au exhibits improved performance, such as high adsorption, superior electro-catalytic activity for direct electrochemistry and promising biocompatibility. ZnO possesses two main peaks that are centered in the UV (~380 nm) and visible regions (500–700 nm). The manifested emission in the UV region occurs via recombination of free excitons. The emission in the visible region corresponds to internal defects, such as oxygen vacancy, zinc vacancy and both oxygen and zinc interstitials. By taking advantage of these basic characteristics, the current study analyzes the ZnO-Au hybrid based on different optical studies and microscopic observations. We also have made an approach to develop an optimized various Au sputtered ZnO film as the critical sensing membrane which greatly determines the sensing performance on Interdigitated Electrode (IDE) sensor, with two electrode (IDE) detection schemes with simple current detection. The novelty of our work is in investigating the correlation between the thicknesses of Au sputtered on the ZnO thin film with its impedance sensing capacity. With the obtained results, an optimized ZnO-Au hybrid thin film is proposed for sensing and other potential applications.

### Morphological features of Au-sputtered ZnO thin film

#### Field emission scanning electron microscopy (FESEM) observations

**Fig 2:**
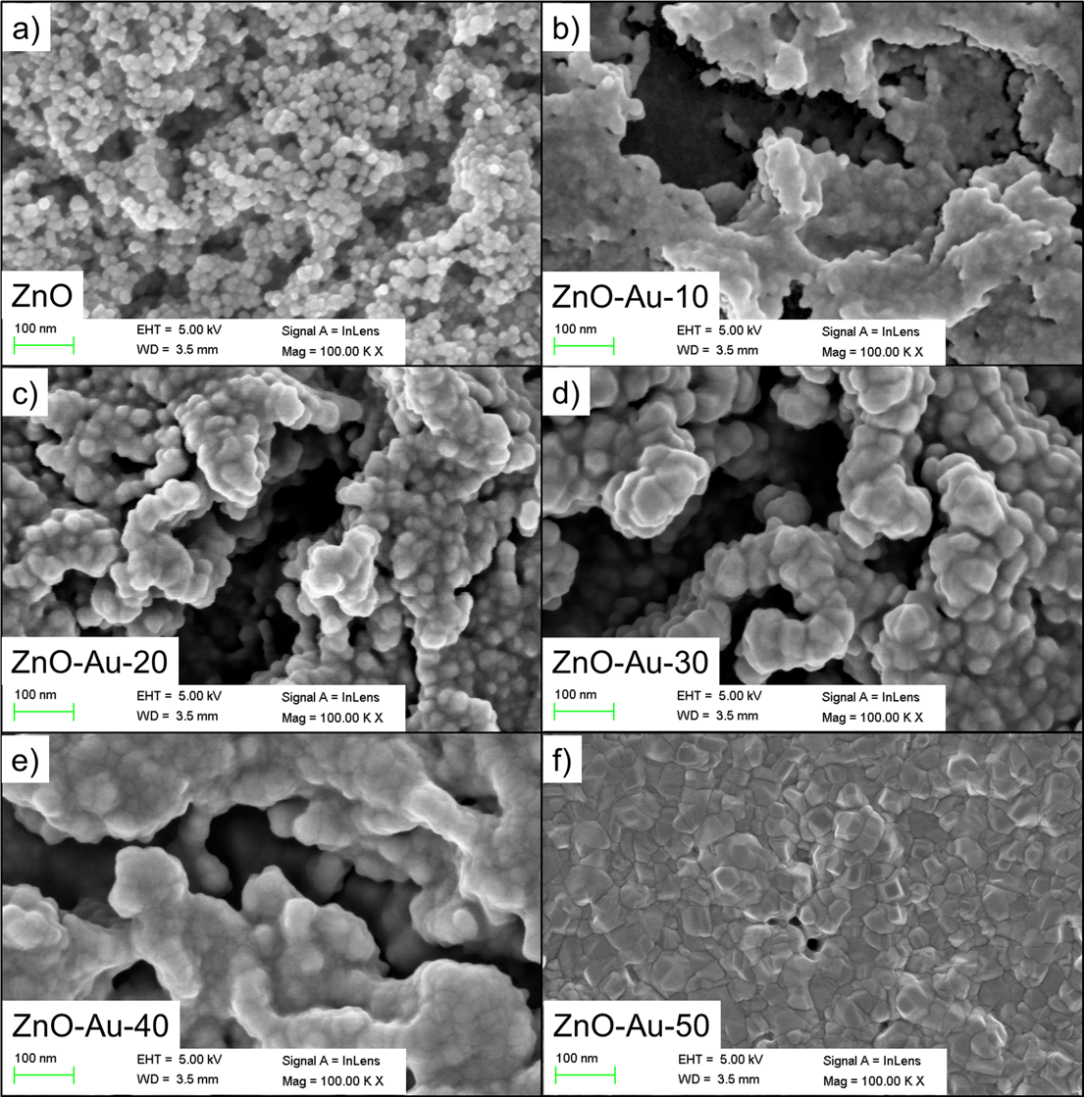
Morphological observation of ZnO thin films sputtered with different thicknesses of Au. Geometrical grain shape analysis and size inspection were conducted to observe the effect of the sputtered thickness of Au. FESEM images of ZnO thin films sputtered with different thicknesses of Au: (a) 0 nm, (b) 10 nm, (c) 20 nm (d) 30 nm, (e) 40 nm and (f) 50 nm.

The morphological features of the as-synthesized ZnO thin film nanostructures sputtered with AuNPs were examined by FESEM and TEM. The results are shown in Fig 2A–2F to illustrate the morphological features of the ZnO thin film obtained before and after deposition of various thicknesses (10–50 nm) of sputtered Au. The FESEM images were visualized with different brightness and contrast due to the presence of the ZnO thin film layer that coated the surface (3 layer coating). A magnified FESEM image is shown in Fig 2A, and this image indicates that the surface of the synthesized ZnO thin films consist of highly crystalline grains with hexagonal nanoparticles. The agglomerates on the observed ZnO thin film prior to Au sputtering has an average diameter of 15 nm over the entire substrate surface, and these agglomerates are compact with a high density. The FESEM images (Fig 2B–2F) confirmed that Au sputtering on the ZnO thin films improved the size of the ZnO nanoparticle as the Au thickness increased. Moreover, the AuNPs changed the hexagonal morphology of the ZnO particle on the surface of the sample to irregularly shaped grains. As the sputtering thickness increased from 40 to 50 nm, the Au-sputtered ZnO thin film surface formed a continuous film due to the accumulation of AuNPs throughout the surface. Agglomerations of AuNPs were observed as the Au thickness increased, demonstrating their homogeneity.

#### Transmission electron microscopy (TEM) observations

**Fig 3:**
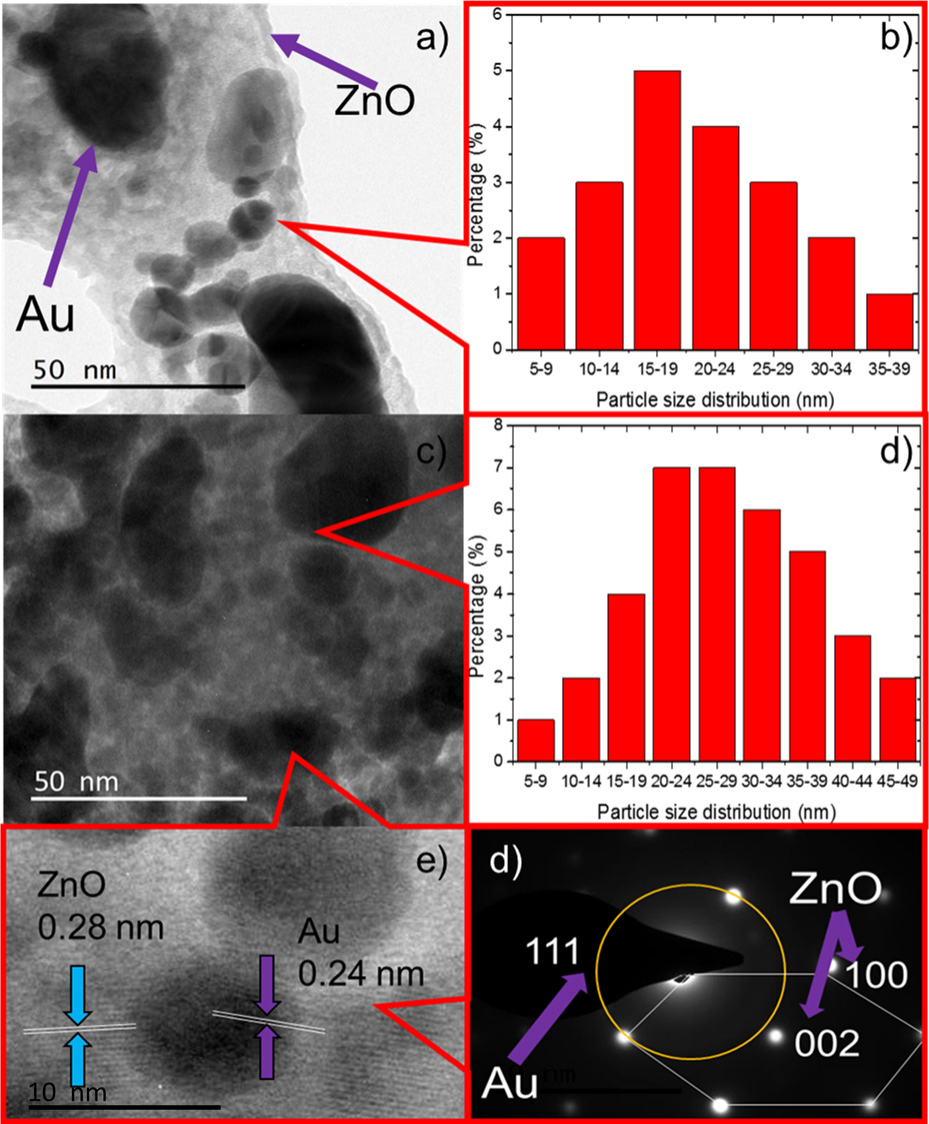
TEM images of the ZnO/Au hybrid. (a) Typical TEM micrograph of 40 nm Au sputtered ZnO. (b) The histogram of 40 nm Au sputtered ZnO showing the particle size distribution. (c) Typical TEM micrograph of 50 nm Au sputtered ZnO. (b) The histogram of 50 nm Au sputtered ZnO showing the particle size distribution. (e) High-resolution TEM image showing the lattice fringes of ZnO and Au. (f) Selected area electron diffraction pattern. ZnO nanowire exhibits a hexagonal spot pattern corresponding to the (100) and (002) planes, and the crystallographic plane of the AuNPs was indexed to (111), corresponding to a hybrid structured Au sputtered ZnO nanocomposite thin film.

The structural morphology of the Au-sputtered ZnO thin film composites was further examined using TEM. The low magnification of TEM of the small fraction/scraps of specimens in Fig 3A and 3C for 40 and 50 nm Au sputtered ZnO provided visualization of both spherical and dumbbell AuNPs structures with dark contrast, and these structures are relatively well deposited onto the highly crystalline surface of the ZnO thin films [37,38]. The AuNPs have a mean diameter of 15–19 nm, with 5 to 39 nm min-max at a sputtered thickness of 40 nm compared to a mean diameter of 20–29 nm, with 5 to 49 nm min-max at a sputtered thickness of 50 nm, respectively (Fig 3B and 3D). AuNPs were seen to be attached on the ZnO thin film structures surfaces; while agglomerating themselves lead to the variation in the mean diameter. The high-resolution transmission electron microscopy (HRTEM) image of the region shown in Fig 3C is shown in Fig 3E. This image indicates that the average diameter of the AuNPs deposited on the ZnO thin films is approximately 10 nm and the AuNPs are dispersed homogenously on the surface of film. In Fig 3E, the observed interplanar lattice fringes of the Au sputtered ZnO thin film owing to the interplanar lattice spacing of 0.24 nm and 0.28 nm corresponding to the spacing for Au (111) crystal planes (FCC) and ZnO (100) planes (Wurtzite), respectively [39–41]. Fig 3F shows the corresponding SAED pattern of the Au-sputtered ZnO thin film, which indicates the presence of a highly crystalline ZnO wurtzite structure and the crystallographic plane of face center cubic AuNPs [42,43]. The SAED patterns further suggested that face center cubic Au were co-exist with wurtzite ZnO nanostructure.

#### Atomic force microscopy (AFM) observations

**Fig 4:**
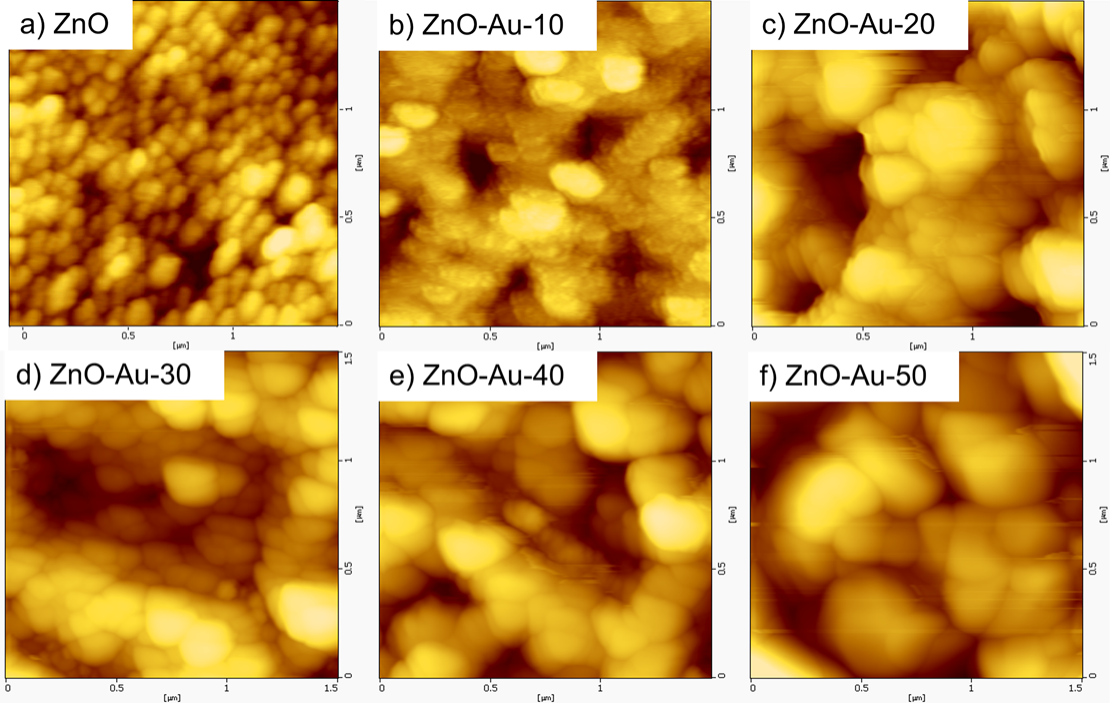
Surface analysis of Au sputtered ZnO. The surface uniformity and roughness were investigated by AFM. 2D AFM images of the ZnO thin films sputtered with different thicknesses of Au: (a) 0 nm, (b) 10 nm, (c) 20 nm (d) 30 nm, (e) 40 nm and (f) 50 nm.

**Fig 5:**
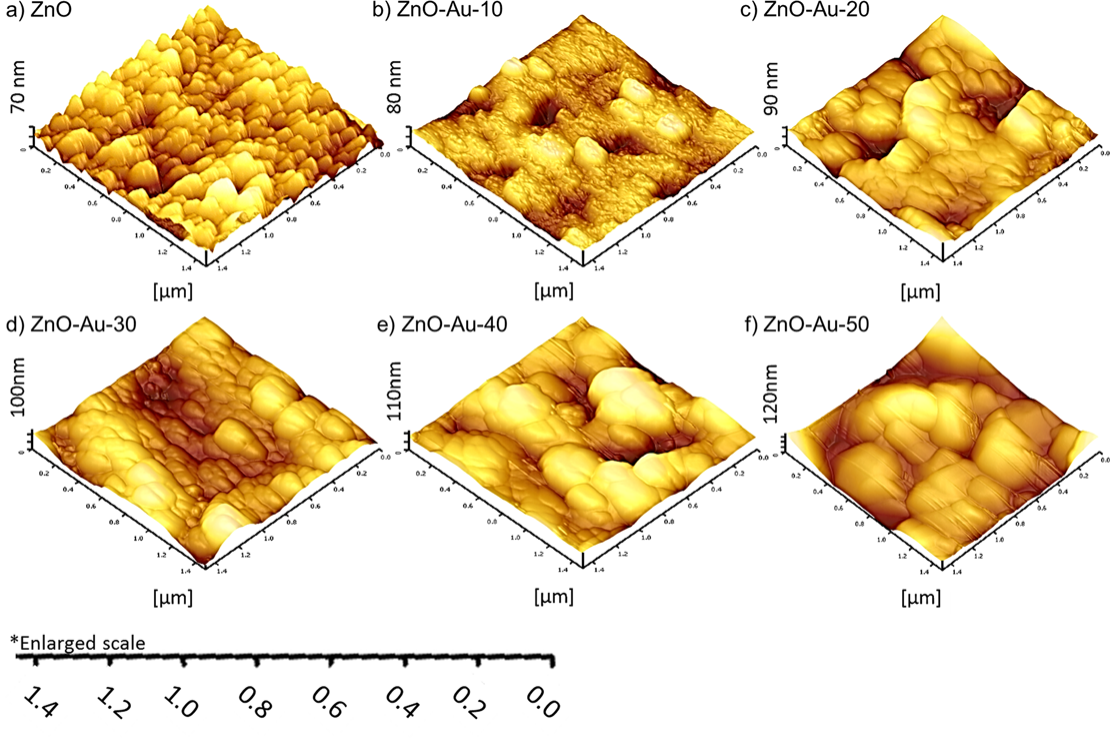
Thickness measurements of Au sputtered ZnO thin films with different thicknesses of sputtering. 3D AFM image of Au sputtered ZnO surface sputtered with different thicknesses of Au: (a) 0 nm, (b) 10 nm, (c) 20 nm (d) 30 nm, (e) 40 nm and (f) 50 nm.

The detailed morphology and surface roughness of the Au-sputtered ZnO thin film were examined by AFM. The AFM measurements were recorded from three different positions with a scanned area of 1.5 x 1.5 μm. Fig 4A–4F show the two-dimensional AFM image obtained for the nanometer scaled ZnO thin film with different thicknesses (0–50 nm) of Au. The results indicate that various thicknesses of Au in the preparation of the Au-sputtered ZnO thin film strongly affect the morphological properties of the ZnO thin film. The AFM micrograph (Fig 4A) displayed a uniform distribution of a granular ZnO grain structure which is consistent with a high-quality ZnO thin film nanostructure with a pin-hole free, smooth and crack-free surface [44,45]. The surface of the films consisted of many granular particles with regular sizes, and the particle/crystallite size increased as the sputtering thickness increased. Fig 5A–5F shows 3D images of bare ZnO and Au sputtered ZnO thin film sample that underwent various Au sputtering thicknesses. When the Au sputtering on ZnO thin films substrate increased from 10 to 50 nm, the overall thicknesses of the thin film were also increased. Fig 5A–5F also shows when Au sputtering on ZnO thin film was increased, the overall surface roughness increased. The grain sizes of the Au-sputtered ZnO nano thin film are listed in Table 1. The grain size increased as the Au-sputtered thickness increased. Therefore, an increase in the Au content on the film surface due to sputtering increased the overall crystallite size, which was confirmed by the XRD analysis. The RMS surface roughness values of these films are 8.4, 15.1, 21.9, 24.0, 32.3 and 37.3 nm [46,47]. An increase in the RMS surface roughness value was observed upon Au sputtering due to the increment in the grain size. As the grain size increased, the surface of the thin film formed island-shaped structures that led to a rougher surface [46,48]. The formation of a large island structure may be due to the sputtered AuNPs, which coalescence with the bipolar ZnO thin film. An increase in the surface roughness of the thin film is associated with a large volume to size ratio, which is favorable for bio-analytical sensing applications.

**Table 1:** Summary of crystallite sizes estimated from the broadening of X-ray diffraction peaks, AFM grain size and RMS value. Optical band gap and calculated refractive indices of Au-sputtered ZnO thin films at different current densities corresponding to the optical dielectric constant.

Sample	Crystallite size (nm)	Grain size (nm)	RMS(nm)	Optical properties		
	XRD	AFM	AFM	Bandgap (eV)	Refractive index (n)	Optical constant (ε_∞_)
**ZnO**	23	30.78	8.35	3.25	2.033^a^; 2.277^b^; 2.319^c^	4.133^a^; 5.185^b^; 5.378^c^
**ZnO/Au 10**	29	62.95	15.05	3.23	2.045^a^; 2.282^b^; 2.323^c^	4.184^a^; 5.207^b^; 5.396^c^
**ZnO/Au 20**	35	96.72	21.97	3.22	2.052^a^; 2.285^b^; 2.325^c^	4.209^a^; 5.221^b^; 5.406^c^
**ZnO/Au 30**	43	125.60	24.01	3.20	2.064^a^; 2.290^b^; 2.330^c^	4.260^a^; 5.244^b^; 5.429^c^
**ZnO/Au 40**	50	157.62	32.30	3.18	2.076^a^; 2.296^b^; 2.334^c^	4.311^a^; 5.272^b^; 5.447^c^
**ZnO/Au 50**	59	184.86	37.29	3.21	2.058^a^; 2.288^b^; 2.327^c^	4.235^a^; 5.235^b^; 5.415^c^

^a^ Ravindra et al [80].

^b^ Herve and Vandamme [81]

^c^Ghosh et al. [82]

### Structural characterizations

#### Energy-dispersive x-ray spectroscopy (EDX) analysis

**Fig 6:**
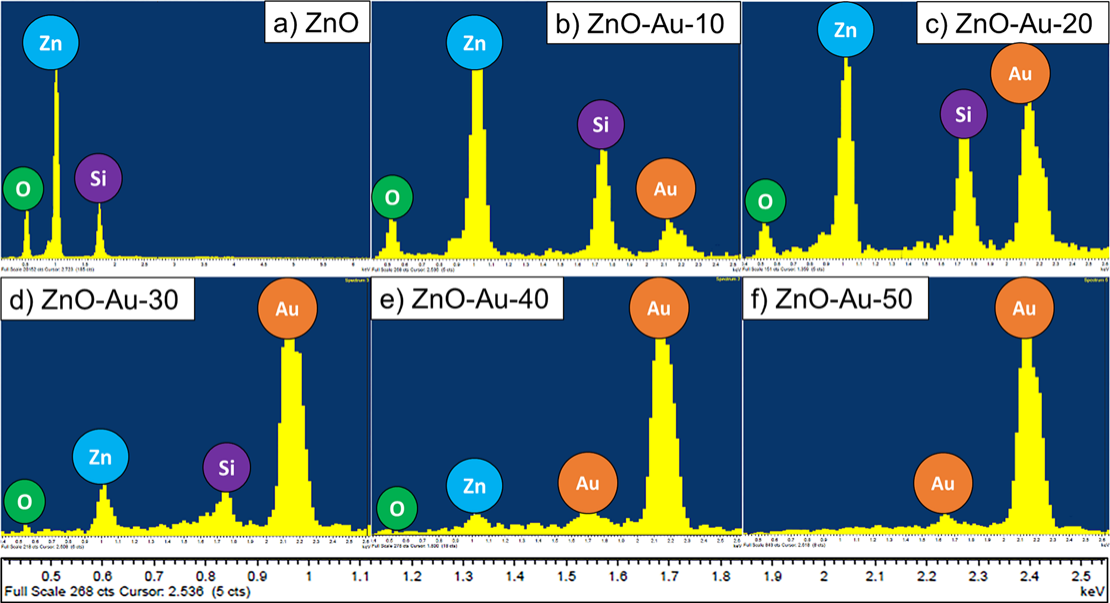
Energy-dispersive X-ray spectra Au sputtered ZnO. The elemental compositions were investigated by EDX. The EDX images of ZnO thin films sputtered with different thicknesses of Au (a) 0 nm, (b) 10 nm, (c) 20 nm (d) 30 nm, (e) 40 nm and (f) 50 nm.

The EDX spectrum of the fabricated Au-sputtered ZnO thin film is shown in Fig 6A–6F. The EDX spectrum confirms the presence of oxygen (O) and zinc (Zn) in the pure ZnO sample, as shown in Fig 6A. The Au peak along with the Zn and O peaks in the Au-sputtered ZnO thin film increased as the doping thickness varied from 10 to 40 nm (Fig 6B–6E). However, when the doping increased to 50 nm thickness, the Zn peak disappeared, thus suggesting the formation of a continuous film due to the accumulation of AuNPs throughout the surface (Fig 6F). Furthermore, this result demonstrates the absence of other impurities in the prepared sample.

#### X-ray diffraction (XRD) analysis

**Fig 7:**
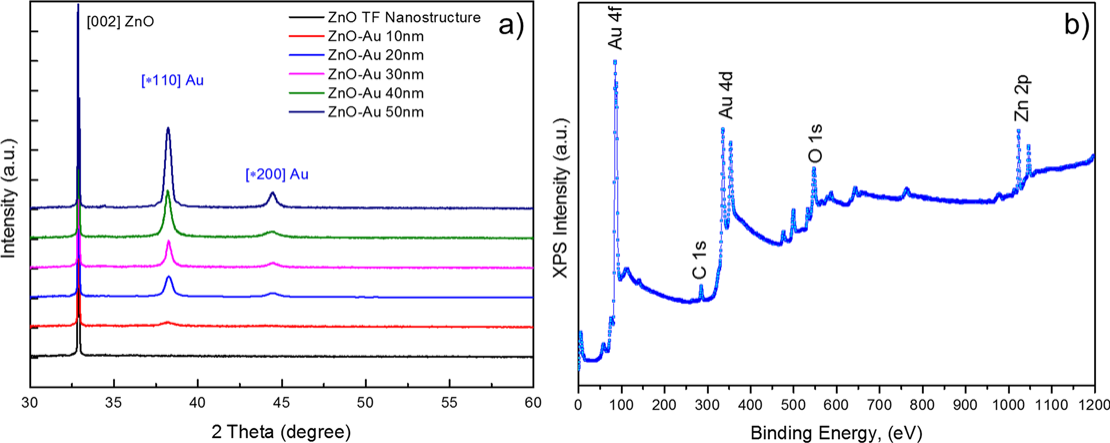
Structural characterizations of Au sputtered ZnO. (a) Structural and grain size analysis of a ZnO thin film matrix. X-ray diffraction analyses of the ZnO thin films sputtered with different thicknesses of Au. (b) Survey scan of the XPS core level spectra for the Au sputtered ZnO nanocomposites. The presence of carbon (C), oxygen (O), zinc (Zn), and gold (Au) were observed in from Au sputtered ZnO thin films without any impurities.

X-ray diffraction analysis was carried out to examine the crystal quality, size, orientation and morphology of the fabricated Au-sputtered ZnO thin film nanostructure. Fig 7A shows a typical XRD pattern of the as-synthesized ZnO thin film coated on the silicon substrate. Fig 7A shows the XRD pattern of the ZnO thin films with various Au thicknesses (10–50 nm). As shown in Fig 7A, the XRD spectra of the Au-sputtered ZnO thin film nanocomposite were compared to that of a pure ZnO thin film. The diffraction peaks corresponding to Au and ZnO were in agreement with those reported in the Joint Committee on Powder Diffraction Standards (JCPDS) reference spectra (No. 36–1451) for standard ZnO and (No. 65–2879) bulk Au, which indicates that the obtain spectra are in good agreement with the standard JCPDS data cards. The obtained XRD patterns exhibited reflection peaks at 32.90° (002). Fig 7Ai can be indexed to the hexagonal wurtzite phase of a ZnO nanostructure [27,49]. Interestingly, two additional diffraction peaks were observed in Fig 7Aii–7Avi compared to the spectra of the pure wurtzite ZnO nanocrystals (Fig 7Ai). The diffraction peaks located at 38.27° and 44.49° were assigned to the (111) and (200) diffraction lines, respectively, which were indexed to the face-centered cubic Au structure [19,50]. This result is in good agreement with the results observed by HRTEM. Here, the (002) reflection appeared to be dominant for all of the samples which suggests that the sol-gel coated ZnO thin film nanostructure preferred anisotropic growth along the [1] direction of the substrate [51]. The observed diffraction peak corresponding to Au (111) and (200) exhibits gradual broadening with sharpening of the increased peak intensity as the Au thickness increased (10 to 50 nm). The Au peak (111), which is shown in Fig 7A, increased in intensity, which indicates significant growth of the Au grain size due to the increasing Au thickness and Ostwald ripening. Ostwald ripening also contributes to the high interatomic forces that occur at larger Au thicknesses [29,52]. The sharp and narrow shape of the peaks indicate that the Au-sputtered ZnO nanocomposites have good crystalline quality. The crystallite domain sizes of the ZnO thin film nanostructure and the AuNPs, which were estimated from the broadening of the XRD peaks for these samples, are listed in Table 1. The obtained FWHM value for the ZnO thin film sputtered with increasing Au thicknesses decreased, which indicates that the crystallinity of the Au-sputtered ZnO thin film improved gradually. Therefore, the grain size of the hybrid ZnO/Au thin film increased when the thin film contained a thicker AuNP layer. As the AuNPs were sputtered onto the ZnO thin film, lattice expansion occurred due to the higher ionic radius of Au (0.85 Å) compared with that of Zn^2+^ (0.74 Å), which resulted in expansion of the lattice bond length and an increase in the grain size of the Au-sputtered ZnO thin films [53,54]. This result indicates that the Au-sputtered ZnO nanocomposites, which were prepared with various Au thicknesses (10–50 nm), exhibited changes in their lattice behavior.

#### X-ray photoelectron spectroscopy (XPS) analysis

**Fig 8:**
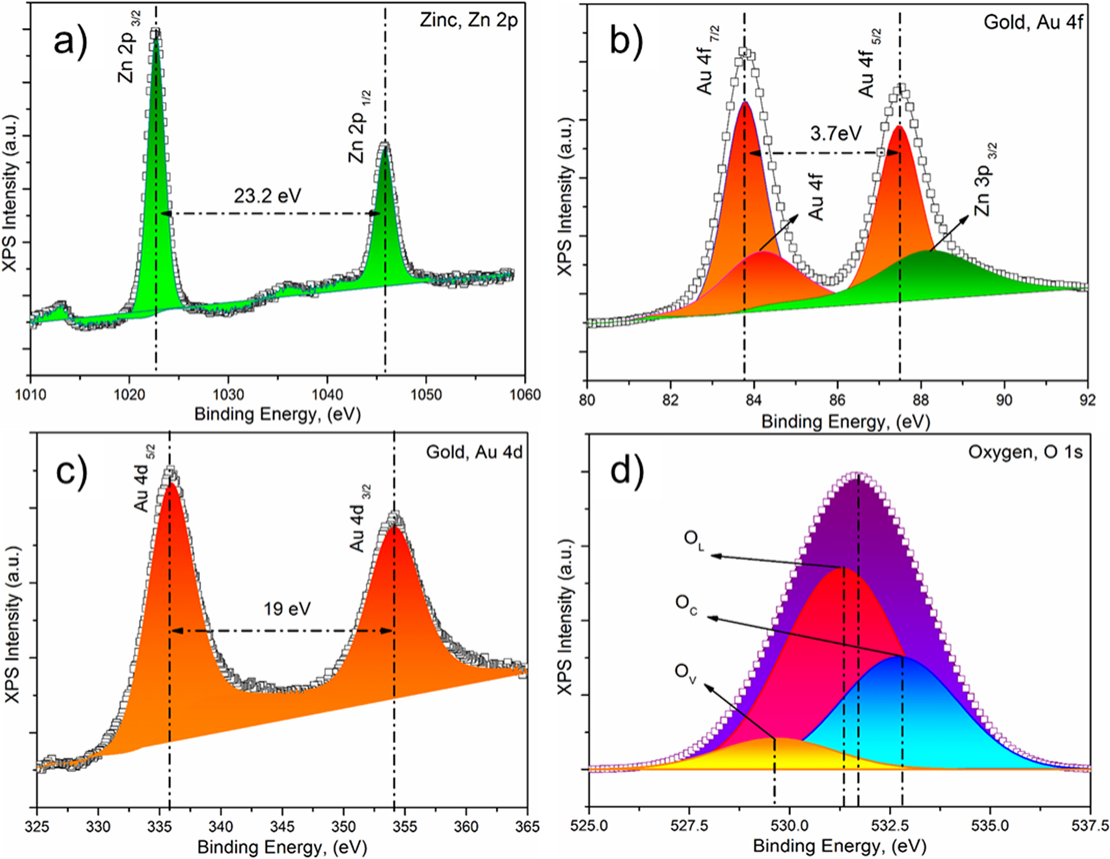
X-ray photoelectron spectroscopy data showing binding energy. (a) Zinc, Zn 2p (b) gold, Au 4f (c) gold, Au 4d and (d) oxygen, O1s electrons.

XPS studies was performed to investigate the elemental composition and chemical state of the elements present in the outmost layer of the Au-sputtered ZnO thin films. The wide XPS survey scan contains photoelectron peaks corresponding to carbon (C), oxygen (O), zinc (Zn), and gold (Au) in the Au-sputtered ZnO thin films without any impurities (Fig 7B). The binding energies are calibrated within an accuracy of 0.1 eV using C1s (284.6 eV). The corresponding O (O1s), Au (Au4d and Au4f) and Zn, Zn2p XPS spectra that were acquired from the Au-sputtered ZnO thin films and the wide scan are shown in Fig 8A–8D. As shown in Fig 8A, the Zn 2p XPS spectra contain two peaks corresponding to Zn 2p_3/2_ and Zn 2p_1/2,_ which correlate to binding energies of 1022.63 eV and 1045.83 eV due to the Zn^2+^ valance state [55–57]. The observed Zn 2p binding energy for the Au-sputtered ZnO thin film exhibits a slightly positive shift of ∆E_zn_ = 1.13 eV compared to the corresponding value for elemental Zn (1021.5 eV) [30,58]. This result indicated that Zn and Au interacted to form a compound due to the structural defects and oxygen vacancies in ZnO. Furthermore, the observed binding energy for Zn 2p revealed the nature of the oxidation state of Zn, and no metallic Zn was observed. Fig 8B shows the core level XPS spectra of Au 4f and Zn 3p. The binding energies at 83.75, 87.45 and 88.2 eV correspond to Au 4f_7/2_, 4f_5/2_ and Zn 3p_3/2_, respectively. The variation in the Au 4f_7/2_ binding energy compared to that of bulk Au (84.0 eV) confirmed the strong electronic interaction between the AuNPs and ZnO [59,60]. Fig 8C shows the core level XPS spectrum of Au 4d for the ZnO/Au-30 thin film. The observed spectrum indicated that the binding energy at 335.87 eV corresponded to spin-orbit spitting of the Au 4d_5/2_ ground state, and the excited state of Au 4d_3/2_, which corresponds to Au^0+^, was observed at approximately 354.09 eV [30]. The core level binding energies corresponding to O1s in the with Gaussian-fitted XPS spectrum for the ZnO/Au-30 thin film are shown in Fig 8D. O1s consists of a broad peak, which was resolved into three peaks located at ~529.56, 531.30 and 532.72 eV. The binding energy peak in the O1s spectrum at 532.72 eV was due to loosely bound oxygen molecules (O_C_) from the ambient atmosphere, which can be easily adsorbed onto the ZnO thin films due to the large surface area–to-volume ratio of the ZnO thin film nanostructures. The peaks centered at 529.56 eV (O_V_) and 531.30 eV (O_L_) correspond to O^2-^ ions on the wurtzite hexagonal structure and oxygen deficient regions within the ZnO thin film matrix [61–64].

### Optical characterizations

#### UV-Vis measurements

**Fig 9:**
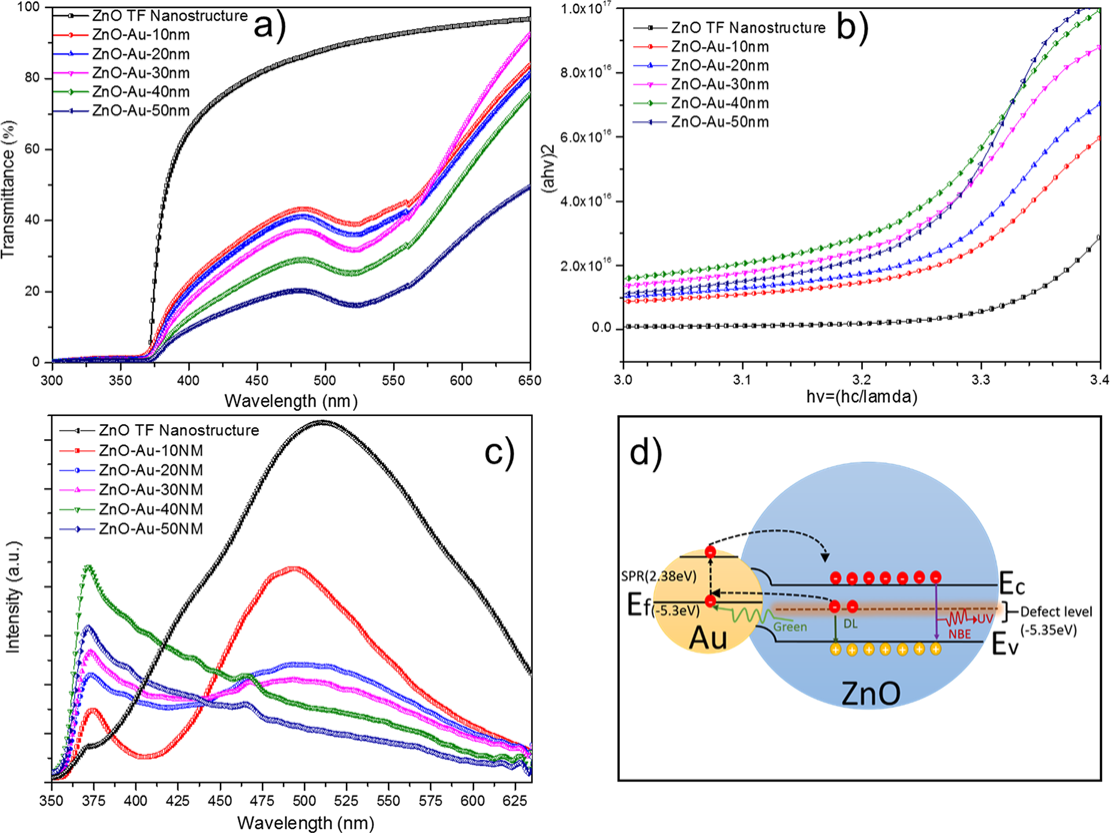
Optical characterizations of Au sputtered ZnO. (a) Au-sputtered ZnO thin films to evaluate their performance for UV transmission applications. (b) Band gap observation of Au-sputtered ZnO nanocomposite thin film using a Tauc plot. (c) Photoluminescence spectra of ZnO thin films with different thicknesses of Au sputtered on the ZnO matrix. (d) Schematic illustration of the SPR effect and energy band alignment on the photoluminescence property of Au-sputtered ZnO nanocomposite with different Au thicknesses.

UV-Vis measurements of the Au-sputtered ZnO thin films provided optical transmission spectra, as shown in Fig 9A. ZnO, which is a semiconductor material with a wide band gap, exhibited a strong transmission at a wavelength of 360–380 nm, as shown in Fig 9A. Nevertheless, sputtering of AuNPs onto the ZnO thin films results in localized surface plasmon resonance (LSPR), which results in another transmission band in the broad spectral range of 400–600 nm [65,66]. If the dimension wavelength of light is larger than that of a metal nanoparticle, an electromagnetic wave is produced with a reverberant coherent oscillation via a particular wavelength. The strong absorption of electromagnetic wave irradiation at a corresponding wavelength resulting from the oscillation is known as SPR [67–69]. Therefore, the peak was intense at a high Au concentration, which can be observed in the absorption curve and can be observed at a wavelength of 510–550 nm in Fig 9A. Moreover, the optical absorption spectra confirmed that the shape of the AuNP is spherical based on the presence of a single peak [67]. According to the spectra shown in Fig 9A, the SPR peak at 510–550 nm displays the partial formation of AuNPs [70,71], since its size increases from 10 to 40 nm caused the SPR trough to get deeper which suggests that at low sizes the NPs are beginning to form, hence it showed partial formation, while at the larger sizes a full formation was seen. As the amount of Au increased, the absorption edge shifted slightly (red shift). Therefore, as the Au thickness increased, the band gap energy of the ZnO thin film decreased due to metal addition [29,67]. The band gap energy for the as-synthesized ZnO and Au-sputtered ZnO sample was measured using a Tauc plot. The absorption coefficient (a) and the photon energy (*hv*) defines the transition state (direct/indirect) during the absorption process. The following equation represents the optical absorption coefficient (a):

$$\text{a}hv=\;D({hv-E_{g})}^{n}$$ (1)

where *D* is a constant, *hv* is the photon energy and *E*_*g*_ is the band gap between the conduction and valence band. The probability of a transition highly influences the n-value, and for a direct transition, the n values are ½ and 3/2. However, for an indirect transition, the n values are 2 and 3 [72,73]. The transition is directly allowed, and the plot of a*hv* as a function of *hv* is linear. Estimation of the band gap energy is achieved by extrapolating the straight-line section to the zero absorption coefficient (a = 0), as shown in Fig 9B. The estimated band gap energies are listed in Table 1. The observed red shift in the spectra was due to AuNP agglomeration and referred to as ‘Localized Surface Plasmon Resonance (LSPR)’. This shift also indicates the strong interfacial coupling between Au and ZnO when Au and ZnO integration occurs. Therefore, electron transfer from Au to ZnO occurs until the two systems achieve equilibrium due to the difference in the work function of both systems. The confined LSPR and the contemporaneous red shift indicate the growth of the AuNPs. Fig 9B is mainly due to the dopant effect of Au. Both the Oxygen (O p) and the Gold (Au d) orbits have t2 symmetry in a tetrahedral environment leading to a strong p-d coupling between them when the Au atom occupies the ZnO site. This moved O 2p level up narrowed the band gap than that of undoped ZnO. As shown in Fig 9B, the dopant effect of Au is the main factor, and the band edge of ZnO/Au is smaller than that of ZnO. The similar result was observed by Naseri et al. and Tarwal et al., shown the decrease in the band gap of the hybrid metal/semiconductor composite resultant from the addition of metal dopant.

The measured band gap value of ZnO with Au incorporated is superior compared to that of the as-synthesized ZnO. For ZnO semiconductors, the conduction band curves upwards, and the valence band curves downwards. For an undoped material, the Fermi level is located in the middle of the band gap. The donor states are formed just below the conduction band when metal dopants are added. Similarly, in this study, Au from sputtering with an increasing thickness from 10 to 40 nm exhibited a typical conduction band that curved upwards and a typical valence band that curved downwards. Therefore, under irradiation with a longer wavelength and with a smaller band gap, the electron-hole pair can be promoted. However, when the metal doping was higher than 40 nm, the opposite shifts were observed (i.e., the conduction band curved downwards and the valence band curved upwards) because the free electron properties are manifested [67,74]. Assuming an increase in the carrier concentration, the Burstein-Moss effect occurs, which explains the phenomenon where the conduction band becomes populated because its lowest states are blocked [75–77]. The apparent band gap of a semiconductor increases as the absorption edge is pushed to high energies, and the electron distribution becomes degenerate in a highly doped semiconductor, which acts like a metal. Based on the number of free electrons donated by the 50 nm thick Au film to ZnO; a sufficiently high number to cause the ZnO Fermi level to shift higher into the conduction band, thus exhibiting a band gap widening i.e. Burstein-Moss effect [78,79]. Comparatively, below 50 nm thickness the ZnO/Au hybrid is not a complete film but instead nanoparticles which limit the number of available charge carriers. These samples showed a normal band gap narrowing with increasing Au thickness, to a maximum of 40 nm. This was further proved by FESEM image illustrating the formation of continuous film of AuNPs covering the surface of the ZnO thin film as the sputtering thickness increased to 50 nm. Thus, the deterioration of the optical properties for 50 nm Au sputtering sample can be justified as Burstein-moss effect.

The refractive indices of the end-point compounds has been calculated and listed in Table 1. The refractive indices are due to three different calculations as formulated by Ravindran et al. [80], Herve and Vandamme [81], and Ghosh et al. [82] where all three formulations for ‘n’ are different due the use of different approaches. The first one is a direct and linear function of absorption and bandgap, the second is an empirical relation inspired from light refraction and dispersion whilst the third is a modified version of the empirical second formulation by taking account of band structure and the quantum dielectric approaches. Pending further experimental confirmation on the exact value of ‘n’ for this hybrid semiconductor-metal nanoparticle system, all three formulations are being adhered to [20,83].

#### Photoluminescence (PL) measurements

To correlate the optical properties of Au-sputtered ZnO with bare ZnO thin films, PL analyses have been performed. Fig 9C shows the PL characteristics manifested by bare ZnO and the Au-sputtered ZnO thin film. For the bare ZnO nanorods, a weak near band edge UV emission located at 373 nm and a broad, strong green emission peak at located 515 nm were observed. In ZnO, the band edge UV emissions were defined as distinction feature emissions of ZnO nanostructures [84]. On the other hand, the broad hump green emission corresponds to a structural defect, such as a Zn vacancy (Vzn) and/or oxygen vacancy (Vo) [85,86]. When the bare ZnO sample was excited using the 325 nm line of a continuous-wave He–Cd laser, only a few electrons reached the conduction band, and a majority of the electrons were trapped in the defect level. Therefore, the electron in the defect level readily recombined with the holes in the valence band of ZnO to produce a broad hump in the visible emission [87,88]. During the formation of ZnO thin film, the incomplete oxidation of Zn was due to insufficient amount of oxygen diffusion to the lattice lead to the formation of oxygen vacancies and relaxation of Zn interstitials. Therefore, the co-exist oxygen vacancies and Zn interstitials responsible for the broad hump green emissions [84]. Moreover, the intensity of the UV band emission was significantly reduced due to lower recombination of the electron-hole pairs from the conduction band. However, for the Au-sputtered ZnO thin film sample, the band edge emission intensity increased substantially as the sputtering thickness increased from 10 to 40 nm. In contrast, the green emission was significantly suppressed to the noise level as the sputtering thickness increased from 10 to 50 nm. In general, the complete suppression of the green emission and the increased band edge emission are related to the presence of the noble metal (Au) in the semiconducting ZnO structure [28,89]. The energy level of the defect states and the Fermi level of Au are very close to each other. Therefore, the electron from the defect level of ZnO can be transferred to the surface plasmons of Au, which significantly increased the electron density at the surface plasmons of Au. Consequently, the surface plasmons of Au were excited, thereby promoting the electron to a higher energy state than the conduction band of ZnO. Then, these electrons can be easily transferred back to the conduction band of ZnO, which facilitates the radiative recombination of the electron-hole pairs in the ZnO valance band [90–92]. Therefore, the recombination between redirected electrons resulting in the enhancement of band-edge emissions intensity of Au-sputtered ZnO thin film sample. The schematic illustration in Fig 9D shows the SPR mechanism of the photoluminescence property with incorporation of a metal nanoparticle, such as Au. However, as the sputtering thickness increased from 10 to 50 nm, the Au particles increased in size and formed a continuous film. In this case, the adsorption process dominated over the scattering process. Therefore, radiation of photons into free space was suppressed due to the non-radiative dispersion of the surface plasmon [23]. These indicate the removal of energy by surface plasmon effect that acts by transferring the energy into other forms but not electromagnetic, hence undetectable as a radiation. This phenomenon accounts for the attenuation of the light emission for ZnO/Au containing a 50-nm- thick AuNP layer.

### Electrical characterizations

#### Impedance analysis

**Fig 10:**
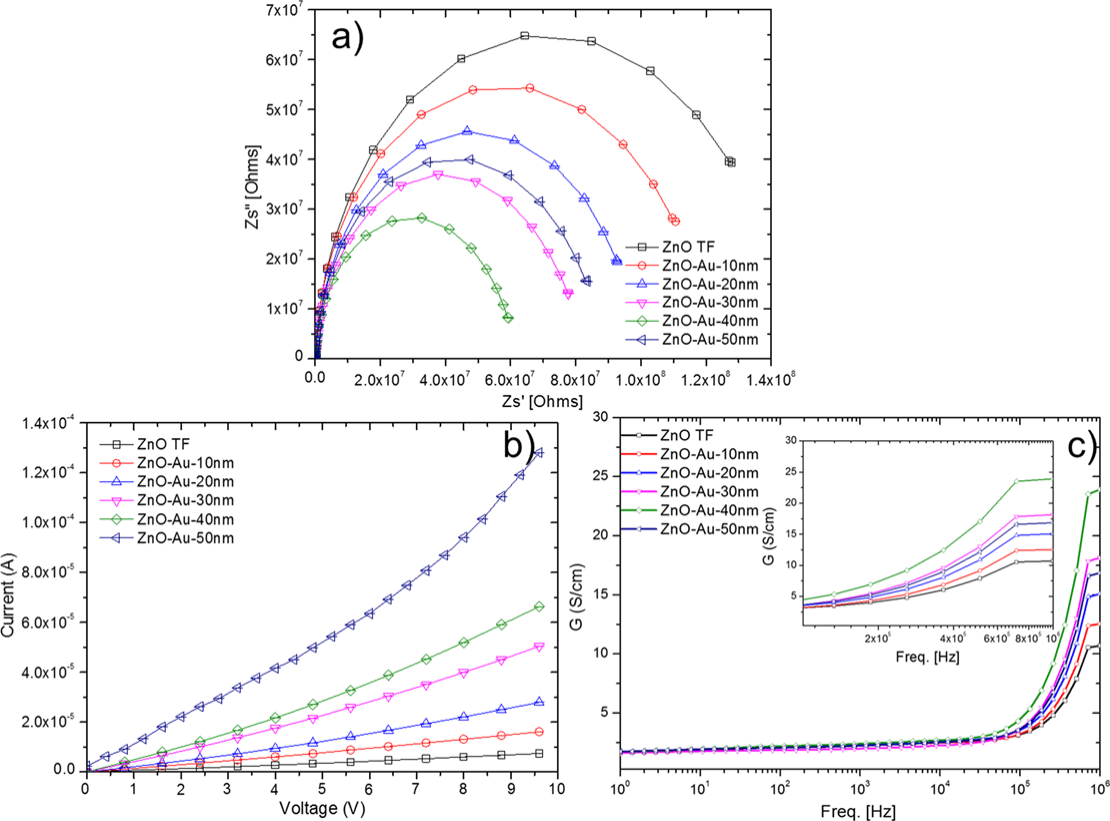
Electrical characterizations of Au sputtered ZnO. (a) Impedance spectra, (b) current to voltage (IV) behavior and (c) AC conduction plot of Au-sputtered ZnO thin films with different Au thicknesses.

The effect of Au thickness onto ZnO film on impedance sensing performances has neither been characterized nor experimentally proven. Hence, to investigate the effect of the sputtered AuNP layers on the conduction mechanism, AC impedance spectroscopic analyses were performed. Fig 10A shows the Nyquist plot, which consists of the real (Z’) and imaginary (Z”) parts of the complex impedance spectra for the Au-sputtered ZnO thin films. These plots represent the relaxation times and resistance related to the bulk grain, grain boundaries and electrode interfaces in the complex impedance plane. As shown in Fig 10A, the total impedance (semicircle arc) of the film decreased as the thickness of the AuNP sputtering increased (0, 10, 20, 30 and 40 nm). These variations were due to the grain sizes and dipole dynamics [93,94]. During the annealing treatment, oxygen molecules from the ambient atmosphere can be easily adsorbed onto the ZnO thin films due to the large surface area–to-volume ratio of the ZnO thin film. These adsorbed oxygen molecules trap many electrons from the conduction band of ZnO at grain boundary surfaces, which leads to an increase in the resistance. Therefore, sufficient numbers of trapped electrons give rise to a strong electric field, which affects the dipoles around the trap site resulting in the formation of spatial charges [95,96]. Therefore, the observed impedance for bare ZnO is the highest. The ZnO thin film that is sputtered with AuNP (10, 20, 30 and 40 nm) is a system in which the Fermi energy level of ZnO is lower than that of Au. This energy-level disparity is due to the work function of ZnO being higher (5.2–5.3 eV) than that of Au (5.1 eV), resulting in an electron transfer from Au to ZnO until the two systems reach dynamic equilibrium [97,98]. The electron transfer leads to an ohmic junction at the agglutination point due to a decrease in the ZnO depletion layer with a higher conductivity, which results in a decrease in the impedance, as shown in Fig 10A [19,99]. However, the opposite trend was observed for the 50 nm thick sputtered AuNP layer. When the sputtering thickness increased to 50 nm, the Au particles grew larger and formed a continuous film, where the ohmic characteristic of the film exhibited rectifying characteristics. In this case, the formation of a continuous film due to the 50 nm thickness led to the formation of a metal-semiconductor junction that forms a Schottky barrier, as shown in the current to voltage (I-V) results in Fig 10B [58,100]. This phenomenon accounts for the attenuated impedance variation for the Au-sputtered ZnO after sputtering of a 50-nm-thick AuNP layer. Further, AC conduction showed a slow increase in conductivity as frequency increases, however, it saturates above 1 MHz, for all thicknesses (Fig 10C). Similarly, saturation plateau increases with Au thickness, up to 40 nm, however, at 50 nm thickness the plateau drops which signify a drop in conductivity, due to a widened bandgap, which makes it difficult for valence to conduction jump for carriers. These results confirm that the 40 nm AuNP coated sensor is the most sensitive with the highest conductance as well as the maximum saturation current. AC conduction plots are in strong agreement with DC IV results, which show an ever linearly increasing trend with voltage. At 50 nm thickness however, the formation of a full film and total coverage of ZnO shows a Schottky behaviour.

## Conclusion

Incorporation of Au into a ZnO thin film resulted in changes in the surface morphology as well as the optical and electrical behavior. The intensity of the surface roughness was enhanced as the hybrid ZnO/Au grain size increased, which was confirmed by AFM observations. Significant difference in the changes in the band gap of hybrid ZnO/Au were observed. The band gap of the ZnO/Au hybrid thin film decreased as the Au thickness increased. Based on the obtained PL results, the peak intensity in the visible region (green emission) decreased when a thicker Au layer was incorporated onto ZnO. Based on the structural, optical and electrical analyses, incorporation of Au substantially improves the host film. However, deterioration in the optical properties of ZnO/Au hybrid thin film was observed when it contained a Au layer that was thicker than 40 nm. In addition, an increase in the thickness of Au (>40 nm) sputtered onto the hybrid ZnO/Au thin film resulted in changes in the electrical performance. The formation of a Schottky barrier when sputtered with a 50 nm thick Au layer alters the n-type conduction nature of ZnO towards p-type conduction based on I-V characterization. In conclusion, our current study highlights the significance of rational engineering of ZnO/Au hybrid structures for use as next generation biosensor systems, which could substantially improve the biomolecule-sensing capabilities. Additional research will provide new avenues of exploration and utilization of robust ZnO/Au hybrid structures to harness their superior optical and electrical properties for detection of biomolecules.

## References

[1] Perumal V, Hashim U. Advances in biosensors : principle, architecture and applications. J Appl Biomed. 2013;11: 1–34.

[2] Perumal V, Hashim U, Gopinath SCB, Haarindraprasad R, Poopalan P, Liu W-W, et al A New Nano-worm Structure from Gold-nanoparticle Mediated Random Curving of Zinc Oxide Nanorods. Biosens Bioelectron. Elsevier; 2015;78: 14–22. 10.1016/j.bios.2015.10.083 26584078

[3] Gopinath SCB, Awazu K, Fujimaki M, Shimizu K, Mizutani W, Tsukagoshi K. Surface functionalization chemistries on highly sensitive silica-based sensor chips. Analyst. 2012;137: 3520 10.1039/c2an35159e 22705905

[4] Yuan YJ, Gopinath SCB, Kumar PKR. Regeneration of commercial Biacore chips to analyze biomolecular interactions. Opt Eng. 2011;50: 034402–1–6. 10.1117/1.3554392

[5] Gopinath SCB, Awazu K, Tominaga J, Kumar PKR. Monitoring biomolecular interactions on a digital versatile disk: A BioDVD platform technology. ACS Nano. 2008;2: 1885–1895. 10.1021/nn800285p 19206429

[6] Nomura K, Gopinath SCB, Lakshmipriya T, Fukuda N, Wang X, Fujimaki M. An angular fluidic channel for prism-free surface-plasmon-assisted fluorescence capturing. Nat Commun. Nature Publishing Group; 2013;4: 2855 10.1038/ncomms3855 24335751

[7] Lakshmipriya T, Fujimaki M, Gopinath SCB, Awazu K. Generation of anti-influenza aptamers using the systematic evolution of ligands by exponential enrichment for sensing applications. Langmuir. 2013;29: 15107–15115. 10.1021/la4027283 24200095

[8] Gopinath SCB, Awazu K, Fujimaki M, Kumar PKR. Signal changes for dye-complexed biomolecular interactions on waveguide-sensor chips. Sensors Actuators, B Chem. 2011;155: 239–244. 10.1016/j.snb.2010.11.054

[9] Fujimaki M, Nomura K, Sato K, Kato T, Gopinath SCB, Wang X, et al Detection of colored nanomaterials using evanescent field-based waveguide sensors. Opt Express. 2010;18: 15732–15740. 10.1364/OE.18.015732 20720956

[10] Jiang R, Li B, Fang C, Wang J. Metal/semiconductor hybrid nanostructures for plasmon-enhanced applications. Adv Mater. 2014;26: 5274–5309. 10.1002/adma.201400203 24753398

[11] Wang X, Zhang X, Cheng W, Shao H, Liu X, Li X, et al Facile synthesis and optical properties of polymer-laced ZnO-Au hybrid nanoparticles. Nanoscale Res Lett. 2014;9: 1–7. 10.1186/1556-276X-9-109 24606946 PMC3973971

[12] Cheng C, Amini A, Zhu C, Xu Z, Song H, Wang N. Enhanced photocatalytic performance of TiO2-ZnO hybrid nanostructures. Sci Rep. 2014;4: 4181 10.1038/srep04181 24566978 PMC3933911

[13] Shi X, Gu W, Li B, Chen N, Zhao K, Xian Y. Enzymatic biosensors based on the use of metal oxide nanoparticles. Microchim Acta. 2013;181: 1–22. 10.1007/s00604-013-1069-5

[14] Balakrishnan SR, Hashim U, Gopinath SCB, Poopalan P, Ramayya HR, Iqbal Omar M, et al A Point-of-Care Immunosensor for Human Chorionic Gonadotropin in Clinical Urine Samples Using a Cuneated Polysilicon Nanogap Lab-on-Chip. PLoS One. 2015;10: e0137891 10.1371/journal.pone.0137891 26368287 PMC4569379

[15] Kumar M, Reddy GB. Ag:ZrO 2 nanocomposite thin films derived using a novel sol-gel technique. Phys Status Solidi. 2009;246: 2232–2237. 10.1002/pssb.200844464

[16] Mishra YK, Adelung R, Röhl C, Shukla D, Spors F, Tiwari V. Virostatic potential of micro-nano filopodia-like ZnO structures against herpes simplex virus-1. Antiviral Res. Elsevier B.V.; 2011;92: 305–312. 10.1016/j.antiviral.2011.08.017 21893101 PMC4148000

[17] Antoine TE, Mishra YK, Trigilio J, Tiwari V, Adelung R, Shukla D. Prophylactic, therapeutic and neutralizing effects of zinc oxide tetrapod structures against herpes simplex virus type-2 infection. Antiviral Res. Elsevier B.V.; 2012;96: 363–375. 10.1016/j.antiviral.2012.09.020 23047013 PMC3535478

[18] Mishra YK, Kaps S, Schuchardt A, Paulowicz I, Jin X, Gedamu D, et al Versatile fabrication of complex shaped metal oxide nano-microstructures and their interconnected networks for multifunctional applications. KONA Powder Part J. 2014;31: 92–110. 10.14356/kona.2014015

[19] Perumal V, Hashim U, Gopinath SCB, Haarindraprasad R, Foo KL, Balakrishnan SR, et al “Spotted Nanoflowers”: Gold-seeded Zinc Oxide Nanohybrid for Selective Bio-capture. Sci Rep. Nature Publishing Group; 2015;5: 12231 10.1038/srep12231 26178973 PMC4503952

[20] Haarindraprasad R, Hashim U, Gopinath SCB, Kashif M, Veeradasan P, Balakrishnan SR, et al Low Temperature Annealed Zinc Oxide Nanostructured Thin Film-Based Transducers: Characterization for Sensing Applications. PLoS One. 2015;10: 1–20. 10.1371/journal.pone.0132755 PMC4500498 26167853

[21] Mishra YK, Modi G, Cretu V, Postica V, Lupan O, Reimer T, et al Direct Growth of Freestanding ZnO Tetrapod Networks for Multifunctional Applications in Photocatalysis, UV Photodetection, and Gas Sensing. ACS Appl Mater Interfaces. 2015;7: 14303–14316. 10.1021/acsami.5b02816 26050666

[22] Gedamu D, Paulowicz I, Kaps S, Lupan O, Wille S, Haidarschin G, et al Rapid fabrication technique for interpenetrated ZnO nanotetrapod networks for fast UV sensors. Adv Mater. 2014;26: 1541–1550. 10.1002/adma.201304363 24249633

[23] Cheng CW, Sie EJ, Liu B, Huan CH a, Sum TC, Sun HD, et al Surface plasmon enhanced band edge luminescence of ZnO nanorods by capping Au nanoparticles. Appl Phys Lett. 2010;96: 3–5. 10.1063/1.3323091

[24] Upadhyayula VKK. Functionalized gold nanoparticle supported sensory mechanisms applied in detection of chemical and biological threat agents: A review. Anal Chim Acta. Elsevier B.V.; 2012;715: 1–18. 10.1016/j.aca.2011.12.008 22244163

[25] Lim Z-ZJ, Li J-EJ, Ng C-T, Yung L-YL, Bay B-H. Gold nanoparticles in cancer therapy. Acta Pharmacol Sin. Nature Publishing Group; 2011;32: 983–990. 10.1038/aps.2011.82 21743485 PMC4002534

[26] Jain PK, El-Sayed IH, El-Sayed MA. Au nanoparticles target cancer. nanotoday. 2007;2: 18–29.

[27] Su L, Qin N. A facile method for fabricating Au-nanoparticles-decorated ZnO nanorods with greatly enhanced near-band-edge emission. Ceram Int. 2015;41: 2673–2679. 10.1016/j.ceramint.2014.10.081

[28] Park S, Mun Y, An S, In Lee W, Lee C. Enhanced photoluminescence of Au-functionalized ZnO nanorods annealed in a hydrogen atmosphere. J Lumin. 2014;147: 5–8. 10.1016/j.jlumin.2013.10.044

[29] Tarwal NL, Devan RS, Ma YR, Patil RS, Karanjkar MM, Patil PS. Spray deposited localized surface plasmonic Au-ZnO nanocomposites for solar cell application. Electrochim Acta. 2012;72: 32–39. 10.1016/j.electacta.2012.03.135

[30] Xu Y, Yao B, Li YF, Ding ZH, Li JC, Wang HZ, et al Chemical states of gold doped in ZnO films and its effect on electrical and optical properties. J Alloys Compd. 2014;585: 479–484. 10.1016/j.jallcom.2013.09.199

[31] Jin Z, Gao L, Zhou Q, Wang J. High-performance flexible ultraviolet photoconductors based on solution-processed ultrathin ZnO/Au nanoparticle composite films. Sci Rep. 2014;4: 4268 10.1038/srep04268 24589625 PMC3940971

[32] Zhang Y, Li X, Ren X. Effects of localized surface plasmons on the photoluminescence properties of Au-coated ZnO films. Opt Express. 2009;17: 8735–8740. 10.1364/OE.17.008735 19466122

[33] Ozga K, Kawaharamura T, Ali Umar A, Oyama M, Nouneh K, Slezak A, et al Second order optical effects in Au nanoparticle-deposited ZnO nanocrystallite films. Nanotechnology. 2008;19: 185709–1–6. 10.1088/0957-4484/19/18/185709 21825705

[34] Balakrishnan SR, Hashim U, Letchumanan GR, Kashif M, Ruslinda a. R, Liu WW, et al Development of highly sensitive polysilicon nanogap with APTES/GOx based lab-on-chip biosensor to determine low levels of salivary glucose. Sensors Actuators A Phys. Elsevier B.V.; 2014;220: 101–111. 10.1016/j.sna.2014.09.027

[35] Balakrishnan SR, Hashim U, Gopinath SCB, Poopalan P, Ramayya HR, Veeradasan P, et al Polysilicon Nanogap Lab-on-Chip Facilitates Multiplex Analyses with Single Analyte. Biosens Bioelectron. Elsevier; 2015; 10.1016/j.bios.2015.10.075 26560969

[36] Perumal V, Prasad RH, Hashim U. pH Measurement using in house fabricated interdigitated capacitive transducer. RSM 2013 IEEE Reg Symp Micro Nanoelectron. Ieee; 2013; 33–36. 10.1109/RSM.2013.6706466

[37] Strunk J, Kähler K, Xia X, Comotti M, Schüth F, Reinecke T, et al Au/ZnO as catalyst for methanol synthesis: The role of oxygen vacancies. Appl Catal A Gen. 2009;359: 121–128. 10.1016/j.apcata.2009.02.030

[38] Mohapatra S, Mishra YK, Ghatak J, Avasthi DK. In-situ tem observation of electron irradiation induced shape transition of elongated gold nanoparticles embedded in silica. Adv Mater Lett. 2013;4: 444–448. 10.5185/amlett.2012.ib.111

[39] Kumar R, Rana D, Umar A, Sharma P, Chauhan S, Chauhan MS. Ag-doped ZnO nanoellipsoids: Potential scaffold for photocatalytic and sensing applications. Talanta. Elsevier; 2015;137: 204–213. 10.1016/j.talanta.2015.01.039 25770626

[40] Song J, Zeng H. Transparent Electrodes Printed with Nanocrystal Inks for Flexible Smart Devices. Angew Chemie Int Ed. Wiley Online Library; 2015;54: 9760–9774. 10.1002/anie.201501233 26223702

[41] Song J, Kulinich SA, Li J, Liu Y, Zeng H. A General One-Pot Strategy for the Synthesis of High-Performance Transparent-Conducting-Oxide Nanocrystal Inks for All-Solution-Processed Devices. Angew Chemie Int Ed. 2014;54: 462–466. 10.1002/anie.201408621 25403980

[42] Mishra YK, Chakravadhanula VSK, Hrkac V, Jebril S, Agarwal DC, Mohapatra S, et al Crystal growth behaviour in Au-ZnO nanocomposite under different annealing environments and photoswitchability. J Appl Phys. 2012;112: 064308–1–5. 10.1063/1.4752469

[43] Wu J-J, Tseng C-H. Photocatalytic properties of nc-Au/ZnO nanorod composites. Appl Catal B Environ. 2006;66: 51–57. 10.1016/j.apcatb.2006.02.013

[44] Saif a. a., Poopalan P. Electrical properties of metal-ferroelectric-insulator-semiconductor structure using BaxSr1-xTiO3 for ferroelectric-gate field effect transistor. Solid State Electron. 2011;62: 25–30. 10.1016/j.sse.2011.03.004

[45] Foo KL, Kashif M, Hashim U, Liu W-W. Effect of different solvents on the structural and optical properties of zinc oxide thin films for optoelectronic applications. Ceram Int. Elsevier; 2014;40: 753–761. 10.1016/j.ceramint.2013.06.065

[46] Kang DJ, Kim JS, Jeong SW, Roh Y, Jeong SH, Boo JH. Structural and electrical characteristics of R.F. magnetron sputtered ZnO films. Thin Solid Films. 2005;475: 160–165. 10.1016/j.tsf.2004.07.029

[47] Mishra YK, Mohapatra S, Kabiraj D, Tripathi a, Pivin JC, Avasthi DK. Growth of Au nanostructures by annealing electron beam evaporated thin films. J Opt A Pure Appl Opt. 2007;9: S410–S414. 10.1088/1464-4258/9/9/S21

[48] Kumar M, Sandeep CSS, Kumar G, Mishra YK, Philip R, Reddy GB. Plasmonic and Nonlinear Optical Absorption Properties of Ag: ZrO2 Nanocomposite Thin Films. Plasmonics. 2014;9: 129–136. 10.1007/s11468-013-9605-z

[49] Tan ST, Chen BJ, Sun XW, Fan WJ, Kwok HS, Zhang XH, et al Blueshift of optical band gap in ZnO thin films grown by metal-organic chemical-vapor deposition. J Appl Phys. 2005;98: 013505–1–5. 10.1063/1.1940137

[50] Guo J, Zhang J, Zhu M, Ju D, Xu H, Cao B. Sensors and Actuators B : Chemical High-performance gas sensor based on ZnO nanowires functionalized by Au nanoparticles. Sensors Actuators B Chem. 2014;199: 339–345.

[51] Kahraman S, Çakmak HM, Çetinkaya S, Bayansal F, Çetinkara H a., Güder HS. Characteristics of ZnO thin films doped by various elements. J Cryst Growth. 2013;363: 86–92. 10.1016/j.jcrysgro.2012.10.018

[52] Mishra YK, Mohapatra S, Singhal R, Avasthi DK, Agarwal DC, Ogale SB. Au-ZnO: A tunable localized surface plasmonic nanocomposite. Appl Phys Lett. 2008;92: 043107–1–3. 10.1063/1.2838302

[53] Nour ES, Echresh A, Liu X, Broitman E, Willander M, Nur O. Piezoelectric and opto-electrical properties of silver-doped ZnO nanorods synthesized by low temperature aqueous chemical method. AIP Adv. 2015;5: 077163–1–10. 10.1063/1.4927510

[54] Sahu D, Panda NR, Acharya BS, Panda a. K. Enhanced UV absorbance and photoluminescence properties of ultrasound assisted synthesized gold doped ZnO nanorods. Opt Mater (Amst). 2014;36: 1402–1407. 10.1016/j.optmat.2014.03.041

[55] Gogurla N, Sinha AK, Santra S, Manna S, Ray SK. Multifunctional Au-ZnO plasmonic nanostructures for enhanced UV photodetector and room temperature NO sensing devices. Sci Rep. 2014;4: 6483 10.1038/srep06483 25255700 PMC4175732

[56] Tarwal NL, Rajgure a. V, Inamdar a. I, Devan RS, Kim IY, Suryavanshi SS, et al Growth of multifunctional ZnO thin films by spray pyrolysis technique. Sensors Actuators A Phys. 2013;199: 67–73. 10.1016/j.sna.2013.05.003

[57] Kashif M, Ali ME, Ali SMU, Hashim U. Sol–gel synthesis of Pd doped ZnO nanorods for room temperature hydrogen sensing applications. Ceram Int. Elsevier; 2013;39: 6461–6466. 10.1016/j.ceramint.2013.01.075

[58] Shinde SS, Korade a. P, Bhosale CH, Rajpure KY. Influence of tin doping onto structural, morphological, optoelectronic and impedance properties of sprayed ZnO thin films. J Alloys Compd. Elsevier B.V.; 2013;551: 688–693. 10.1016/j.jallcom.2012.11.057

[59] Hosseini ZS, Mortezaali A, Iraji A, Fardindoost S. Sensitive and selective room temperature H2S gas sensor based on Au sensitized vertical ZnO nanorods with flower-like structures. J Alloys Compd. 2015;628: 222–229.

[60] Mohapatra S, Mishra YK, Avasthi DK, Kabiraj D, Ghatak J, Varma S. Synthesis of gold-silicon core-shell nanoparticles with tunable localized surface plasmon resonance. Appl Phys Lett. 2008;92: 103105–1–3. 10.1063/1.2894187

[61] Chen Y, Jyoti N, Hyun-U K, Kim J. Effect of annealing temperature on the characteristics of ZnO thin films. J Phys Chem Solids. Elsevier; 2012;73: 1259–1263. 10.1016/j.jpcs.2012.06.007

[62] Zhang X, Qin J, Xue Y, Yu P, Zhang B, Wang L, et al Effect of aspect ratio and surface defects on the photocatalytic activity of ZnO nanorods. Sci Rep. 2014;4: 4596 10.1038/srep04596 24699790 PMC3975220

[63] Wu A, Jing L, Wang J, Qu Y, Xie Y, Jiang B, et al ZnO-dotted porous ZnS cluster microspheres for high efficient, Pt-free photocatalytic hydrogen evolution. Sci Rep. 2015;5: 8858 10.1038/srep08858 25748688 PMC4352920

[64] Kumar M, Reddy GB. Stability-Inspired Entrapment of Ag Nanoparticles in ZrO2 Thin films. Plasmonics. Plasmonics; 2015; 10.1007/s11468-015-0044-x

[65] Sun Y, Xia Y. Gold and silver nanoparticles: a class of chromophores with colors tunable in the range from 400 to 750 nm. Analyst. 2003;128: 686–691. 10.1039/b212437h 12866889

[66] Deki S, Aoi Y, Yanagimoto H, Ishii K, Akamatsu K, Mizuhata M, et al Preparation and characterization of Au-dispersed TiO2 thin films by a liquid-phase deposition method. J Mater Chem. 1996;6: 1879 10.1039/jm9960601879

[67] Naseri N, Azimirad R, Akhavan O, Moshfegh a. Z. Improved electrochromical properties of sol-gel WO3 thin films by doping gold nanocrystals. Thin Solid Films. 2010;518: 2250–2257. 10.1016/j.tsf.2009.08.001

[68] Kumar M, Reddy GB. Tailoring surface plasmon resonance in Ag : ZrO 2 nanocomposite thin films. Phys E Low-dimensional Syst Nanostructures. 2010; 10.1016/j.physe.2010.08.031

[69] Kumar M, Kulriya PK, Pivin JC, Avasthi DK. Evolution and tailoring of plasmonic properties in Ag:ZrO2 nanocomposite films by swift heavy ion irradiation. J Appl Phys. 2011;109: 044311 10.1063/1.3555593

[70] Sangpour P, Akhavan O, Moshfegh a. Z. rf reactive co-sputtered Au-Ag alloy nanoparticles in SiO2 thin films. Appl Surf Sci. 2007;253: 7438–7442. 10.1016/j.apsusc.2007.03.050

[71] Underwood S, Mulvaney P. Effect of the Solution Refractive Index on the Color of Gold Colloids. Langmuir. 1994;10: 3427–3430. 10.1021/la00022a011

[72] Naseri N, Amiri M, Moshfegh a Z. Visible photoenhanced current–voltage characteristics of Au:TiO2 nanocomposite thin films as photoanodes. J Phys D Appl Phys. 2010;43: 105405–1–8. 10.1088/0022-3727/43/10/105405

[73] Joseph B, Gopchandran KG, Manoj PK, Koshy P, Vaidyan VK. Optical and electrical properties of zinc oxide films prepared by spray pyrolysis. Bull Mater Sci. 1999;22: 921–926. 10.1007/BF02745554

[74] Kim WM, Kim JS, Jeong J, Park J-K, Baik Y-J, Seong T-Y. Analysis of optical band-gap shift in impurity doped ZnO thin films by using nonparabolic conduction band parameters. Thin Solid Films. Elsevier B.V.; 2013;531: 430–435. 10.1016/j.tsf.2013.01.078

[75] Xu J, Shi S, Zhang X, Wang Y, Zhu M, Li L. Structural and optical properties of (Al, K)-co-doped ZnO thin films deposited by a sol–gel technique. Mater Sci Semicond Process. Elsevier; 2013;16: 732–737. 10.1016/j.mssp.2012.12.016

[76] Arshad M, Meenhaz M, Ahmed AS, Tripathi P, Ashraf SSZ, Naqvi AH, et al Band gap engineering and enhanced photoluminescence of Mg doped ZnO nanoparticles synthesized by wet chemical route. J Lumin. 2015;161: 275–280.

[77] Wang FH, Yang CF, Lee YH. Deposition of F-doped ZnO transparent thin films using ZnF2-doped ZnO target under different sputtering substrate temperatures. Nanoscale Res Lett. 2014;9: 1–7. 10.1186/1556-276X-9-97 24572004 PMC3984747

[78] Kumar M, Wen L, Sahu BB, Han JG. Simultaneous enhancement of carrier mobility and concentration via tailoring of Al-chemical states in Al-ZnO thin films. Appl Phys Lett. 2015;106: 241903 10.1063/1.4922732

[79] Kumar M, Kumar T, Avasthi DK. Study of thermal annealing induced plasmonic bleaching in Ag:TiO2 nanocomposite thin films. Scr Mater. 2015;105: 46–49. 10.1016/j.scriptamat.2015.04.030

[80] Notes S, Srivastava VK. On the Perm Gap in Semiconductors. Phys Status Solidi. 1979;93: K115–K160.

[81] Hervé PJL, Vandamme LKJ. Empirical temperature dependence of the refractive index of semiconductors. J Appl Phys. 1995;77: 5476–5477. 10.1063/1.359248

[82] Ghosh D, Samanta L, Bhar G. A simple model for evaluation of refractive indices of some binary and ternary mixed crystals. Infrared Phys. 1984;24: 43–47. Available: http://www.sciencedirect.com/science/article/pii/0020089184900460

[83] Al-Douri Y. Optical Properties of GaN Nanostructures for Optoelectronic Applications. Procedia Eng. 2013;53: 400–404. doi:10.1016/j.proeng.2013.02.052

[84] Zeng H, Duan G, Li Y, Yang S, Xu X, Cai W. Blue Luminescence of ZnO Nanoparticles Based on Non-Equilibrium Processes: Defect Origins and Emission Controls. Adv Funct Mater. 2010;20: 561–572. 10.1002/adfm.200901884

[85] Jin X, Götz M, Wille S, Mishra YK, Adelung R, Zollfrank C. A novel concept for self-reporting materials: Stress sensitive photoluminescence in ZnO tetrapod filled elastomers. Adv Mater. 2013;25: 1342–1347. 10.1002/adma.201203849 23192988

[86] Fabbri F, Villani M, Catellani A, Calzolari A, Cicero G, Calestani D, et al Zn vacancy induced green luminescence on non-polar surfaces in ZnO nanostructures. Sci Rep. 2014;4: 5158 10.1038/srep05158 24894901 PMC5154428

[87] Lai CW, An J, Ong HC. Surface-plasmon-mediated emission from metal-capped ZnO thin films. Appl Phys Lett. 2005;86: 251105–1–3. 10.1063/1.1954883

[88] Lin J-H, Patil R a., Devan RS, Liu Z-A, Wang Y-P, Ho C-H, et al Photoluminescence mechanisms of metallic Zn nanospheres, semiconducting ZnO nanoballoons, and metal-semiconductor Zn/ZnO nanospheres. Sci Rep. 2014;4: 6967 10.1038/srep06967 25382186 PMC4225553

[89] Shao D, Sun H, Yu M, Lian J, Sawyer S. Enhanced Ultraviolet Emission from Poly(vinyl alcohol) ZnO Nanoparticles Using a SiO 2 −Au Core/Shell Structure. Nano Lett. 2012;12: 5840–5844. 10.1021/nl3031955 23094803

[90] Khoa NT, Kim SW, Yoo D-H, Cho S, Kim EJ, Hahn SH. Fabrication of Au/Graphene-Wrapped ZnO-Nanoparticle-Assembled Hollow Spheres with Effective Photoinduced Charge Transfer for Photocatalysis. ACS Appl Mater Interfaces. 2015;7: 3524–3531. 10.1021/acsami.5b00152 25629618

[91] Lin HY, Cheng CL, Chou YY, Huang LL, Chen YF, Tsen KT. Enhancement of band gap emission stimulated by defect loss. Opt Express. 2006;14: 2372–2379. 10.1364/OE.14.002372 19503575

[92] Rouhi J, Mamat MH, Ooi CHR, Mahmud S, Mahmood MR. High-Performance Dye-Sensitized Solar Cells Based on Morphology-Controllable Synthesis of ZnO–ZnS Heterostructure Nanocone Photoanodes. PLoS One. 2015;10: 1–14. 10.1371/journal.pone.0123433 PMC4395432 25875377

[93] Saif AA, Poopalan P. Correlation between the chemical composition and the conduction mechanism of barium strontium titanate thin films. J Alloys Compd. 2011;509: 7210–7215. 10.1016/j.jallcom.2011.04.068

[94] Srivastava S, Srivastava AK, Singh P, Baranwal V, Kripal R, Lee J-H, et al Synthesis of zinc oxide (ZnO) nanorods and its phenol sensing by dielectric investigation. J Alloys Compd. 2015;644: 597–601. 10.1016/j.jallcom.2015.04.220

[95] Kashif M, Ali ME, Ali SMU, Hashim U, Hamid SBA. Impact of hydrogen concentrations on the impedance spectroscopic behavior of Pd-sensitized ZnO nanorods. Nanoscale Res Lett. 2013;8: 1–9. 10.1186/1556-276X-8-68 23399029 PMC3599888

[96] Alaeddin AS, P. P. Impedance/Modulus Analysis of Sol-gel Ba_xSr_1-xTiO_3 Thin Films. J Korean Phys Soc. 2010;57: 1449–1455. 10.3938/jkps.57.1449

[97] Wang X, Kong X, Yu Y, Zhang H. Synthesis and characterization of water-soluble and bifunctional ZnO-Au nanocomposites. J Phys Chem C. 2007;111: 3836–3841. Available: http://pubs.acs.org/doi/abs/10.1021/jp064118z

[98] Shan G, Zhong M, Wang S, Li Y, Liu Y. The synthesis and optical properties of the heterostructured ZnO/Au nanocomposites. J Colloid Interface Sci. 2008;326: 392–5. 10.1016/j.jcis.2008.06.027 18639886

[99] Foo KL, Hashim U, Voon CH, Kashif M, Ali ME. Au decorated ZnO thin film: application to DNA sensing. Microsyst Technol. Springer Berlin Heidelberg; 2015; 1–8. 10.1007/s00542-015-2572-x

[100] Dhara S, Giri PK. On the origin of enhanced photoconduction and photoluminescence from Au and Ti nanoparticles decorated aligned ZnO nanowire heterostructures. J Appl Phys. 2011;110 10.1063/1.3671023

